# In silico optimization of a guava antimicrobial peptide enables combinatorial exploration for peptide design

**DOI:** 10.1038/s41467-018-03746-3

**Published:** 2018-04-16

**Authors:** William F. Porto, Luz Irazazabal, Eliane S. F. Alves, Suzana M. Ribeiro, Carolina O. Matos, Állan S. Pires, Isabel C. M. Fensterseifer, Vivian J. Miranda, Evan F. Haney, Vincent Humblot, Marcelo D. T. Torres, Robert E. W. Hancock, Luciano M. Liao, Ali Ladram, Timothy K. Lu, Cesar de la Fuente-Nunez, Octavio L. Franco

**Affiliations:** 1 Centro de Análises Proteômicas e Bioquímicas, Pós-Graduação em Ciências Genômicas e Biotecnologia Universidade Católica de Brasília, Brasília, 70790-160 DF Brazil; 2 S-Inova Biotech, Pós-graduação em Biotecnologia, Universidade Católica Dom Bosco, Campo Grande, 79117-010 MS Brazil; 3 Porto Reports, Brasília, 72236-011 DF Brazil; 4 Molecular Pathology Post-graduate Program, University of Brasília, Brasília, 70.910-900 DF Brazil; 5 Laboratório de RMN, Instituto de Química, Universidade Federal de Goiás, Goiânia, 74690-900 GO Brazil; 6 Centre for Microbial Diseases and Immunity Research, University of British Columbia, 2259 Lower Mall Research Station, Vancouver, BC V6T 1Z4 Canada; 7 Sorbonne Université, CNRS, Laboratoire de Réactivité de Surface (LRS), UPMC Univ Paris 06, UMR 7197, Paris, F-75005 France; 8 Synthetic Biology Group, MIT Synthetic Biology Center; The Center for Microbiome Informatics and Therapeutics; Research Laboratory of Electronics, Department of Biological Engineering, and Department of Electrical Engineering and Computer Science, Massachusetts Institute of Technology, Cambridge, 02139 MA USA; 9 Broad Institute of MIT and Harvard, Cambridge, 02139 MA USA; 10 Centro de Ciências Naturais e Humanas, Universidade Federal do ABC, Santo André, São Paulo, 09210-580 Brazil; 11 Sorbonne Université, CNRS, Institut de Biologie Paris-Seine (IBPS), Biogenèse des Signaux Peptidiques (BIOSIPE), UPMC Univ Paris 06, Paris, F-75005 France

## Abstract

Plants are extensively used in traditional medicine, and several plant antimicrobial peptides have been described as potential alternatives to conventional antibiotics. However, after more than four decades of research no plant antimicrobial peptide is currently used for treating bacterial infections, due to their length, post-translational modifications or  high dose requirement for a therapeutic effect . Here we report the design of antimicrobial peptides derived from a guava glycine-rich peptide using a genetic algorithm. This approach yields guavanin peptides, arginine-rich α-helical peptides that possess an unusual hydrophobic counterpart mainly composed of tyrosine residues. Guavanin 2 is characterized as a prototype peptide in terms of structure and activity. Nuclear magnetic resonance analysis indicates that the peptide adopts an α-helical structure in hydrophobic environments. Guavanin 2 is bactericidal at low concentrations, causing membrane disruption and triggering hyperpolarization. This computational approach for the exploration of natural products could be used to design effective peptide antibiotics.

## Abstract (web summary)

Antimicrobial peptides are considered promising alternatives to antibiotics. Here the authors developed a computational algorithm that starts with peptides naturally occurring in plants and optimizes this starting material to yield new variants which are highly distinct from the parent peptide.

## Introduction

Hospital-acquired infections are a major global health concern and represent the sixth leading cause of death in the United States, with an estimated cost of ~$10 billion annually^1^. Infections caused by Gram-negative bacteria have been associated with more than 60% of pneumonia cases and more than 70% of urinary tract infections in intensive care units^2^. Besides, such bacteria are highly efficient in generating mutants and sharing genes that encode for mechanisms of antibiotic resistance^1^. It has been recently estimated that 30 million sepsis cases occur worldwide each year, and potentially 5 million deaths occur as a result of antibiotic-resistant infections^3^. Unfortunately, in the past two decades only two classes of antibiotics have reached the market, oxazolidinones and cyclic lipopeptides, and both of these drugs are limited as they only target Gram-positive bacteria^4^. In this context, there is an urgent need to develop alternatives to antibiotics, particularly against Gram-negative bacteria, and apply them as strategies to control bacterial resistance.

 Plants are extensively used in traditional medicine and are a good source of natural products, including antimicrobial peptides (AMPs)^5^. AMPs have been proposed as a promising alternative to conventional antibiotics, and are considered potential next-generation antimicrobial agents^6,7^. However, in more than 40 years of research, no plant AMP has been used to treat bacterial infections in humans, partly due to their limited antimicrobial activity and difficult obtainment using current methods of chemical synthesis^8,9^. Recent advancements in screening methods as well as improved strategies for peptide design^7,10^ could hold promise in the development of plant-derived AMPs, reducing their length or reducing the minimum inhibitory concentrations (MIC).

The AMP rational design methods could be split in two main categories, in cerebro design and computer-aided design^11^, both of which have successfully been used to generate synthetic AMP sequences. However, both strategies are strongly influenced by the information encoded in AMP sequences deposited in databases, which limits their capacity to identify unknown AMP sequences beyond those described in the literature. In cerebro design methods rely on the bacterial membrane as a target for AMPs; in practical terms, this kind of design approach creates and/or modifies peptide sequences by means of increasing peptide cationicity and hydrophobicity, mainly by inserting lysine, leucine, and alanine residues within the sequence, thus enhancing the interaction between peptide and membrane^12,13^. Computer-aided design methods make it possible to explore the peptide sequence space of AMPs using a number of algorithms. Unfortunately, the optimal solutions of such approaches end up sharing approximately 40% identity with AMP sequences deposited in the databases^14–16^, converging on a relatively small portion of AMP sequences composed of a restricted set of amino acids^17,18^. Even when incorporating non-proteinogenic amino acids into AMP sequences, for instance by exchanging cationic or hydrophobic residues for ornithine or norleucine residues, respectively^15,19^, this approach fails to identify AMP sequences with unique amino acid composition.

Thus, known AMPs show redundancy in their primary sequence, suggesting that only a little parcel of the combinatorial sequence space (20^*x*^, *x* being the number of residues in a peptide chain) have been exploited. However, it is possible to identify in nature, mainly in plants, various AMPs with distinct composition, including glycine-rich Pg-AMP1^20^, glycine- and histidine-rich shepherin I and II^21^ or proline-rich *BnPRP1*^22^, representing other parcels of the combinatorial space.

Here we show that these plant sequences can serve as templates to facilitate computer-aided design of synthetic sequences. The guava glycine-rich peptide, Pg-AMP1, is used as a template to generate the guavanin peptides by means of a genetic algorithm with point modifications to design truly innovative peptides, including the application of an equation instead of a machine learning classifier, and the interruption of the algorithm before it reaches a plateau, thus allowing exploration of another parcel of peptide combinatorial sequence space.

## Results

### Design and screening of guavanins

**Fig. 1:**
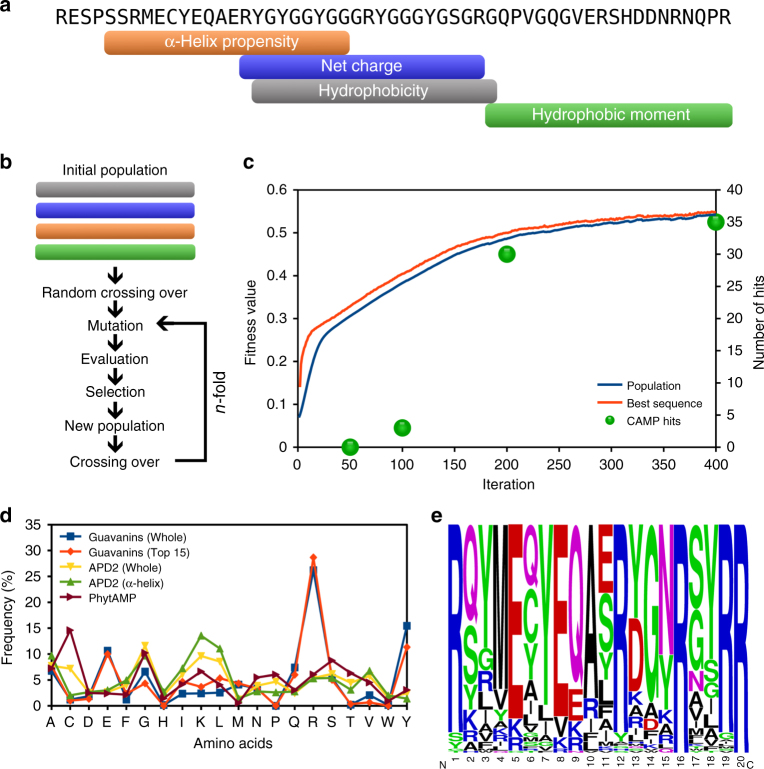
Design and selection of guavanins. **a** Fragment mapping into the Pg-AMP1 sequence. Each fragment represents the maximum value of its respective physicochemical property: in orange, the α-helix propensity (0.553); in blue, the positive net charge (+3); in gray, the average hydrophobicity (−0.092); and in green, the hydrophobic moment (0.3). **b** Flowchart of our custom genetic algorithm. The four Pg-AMP1 fragments were used as the initial population; in the first iteration a totally random sequence selection system for crossing over was applied, in order to improve the diversity of sequences and in the subsequent iterations a roulette wheel selection model was applied for selection of sequences for crossing over. **c** Fitness function evolution during the algorithm iterations and the number of CAMP hits of the highest ranked sequence at iterations 50, 100, 200, and 400; Guavanin sequences were retrieved from the 50th iteration. **d** Amino acid distribution of guavanins and AMPs from APD2 and PhytAMP. Blue squares represent data obtained from 100 guavanin sequences; orange diamonds, the top 15 guavanins; yellow down triangles, the overall APD2 composition; green up triangles, the composition of α-helical peptides from APD2; and brown right triangles the plant AMP sequences from PhytAMP (37). **e** The frequency logo of the 100 generated guavanin sequences (Supplementary Table 1), showing that they are arginine-rich peptides, Arg residues are in at least 20% of their compositions

Overall, genetic algorithms optimize a particular property (the fitness function) from a population of potential solutions (the sequences). Here, the ratio between hydrophobic moment and α-helix propensity was used in the fitness function (see Methods) for selecting amphipathic α-helical peptides, while the initial population consisted of four Pg-AMP1 fragments derived according to specific physicochemical properties (Fig. 1a). One hundred independent simulations of the algorithm were performed, with the parameters set as follows: 250 sequences in the population (generated by random crossing over in the first iteration and fitness guided crossing over in subsequent iterations), 50 with the worst fitness values for discard, single point crossover, and 0.5% of probability of mutation (Fig. 1b). As shown in Fig. 1c, the fitness values for the population and for the best sequence were improved without reaching stabilization and with no hits in CAMP databases, at 50th iteration, indicating a suboptimal solution and a different composition.

The final set was composed of the best sequence of each parallel run, comprising peptides with fitness values ranging from 0.245 to 0.393, named guavanins 1–100 (Supplementary Table 1). The amino acid composition of all the guavanins was different from other AMPs deposited in the Antimicrobial Peptides Database (APD), even taking into account only those peptides assigned with an α-helical structure (Fig. 1d), and despite guavanins are Arg-rich peptides, it draws attention the fact that they use Tyr residues as the hydrophobic counterpart (Fig. 1e).

As we stopped the algorithm before the optimal solution (which would enrich for amino acids present in conventional AMPs), we performed ab initio molecular modeling to verify the α-helical conformation for 15 guavanins with the greatest fitness value, where all of them showed such structure (Supplementary Fig. 1, Supplementary Table 2), indicating that even at suboptimal solutions it is possible to obtain amphipathic α-helices, which is the basis of selection of our fitness function. As the guavanins resemble AMPs, we next  screened their activities against *Pseudomonas aeruginosa* and human erythrocytes, using unpurified peptides generated by SPOT-synthesis on cellulose membranes^23^.

As shown in Supplementary Table 3, none of the 15 guavanins were hemolytic at the highest concentration tested (200 μg mL^−1^), and 8 of them were considered active because their MICs were equal to or lower than the MIC of our positive peptide control, magainin 2 (100 μg mL^−1^). Due to their low MIC values and lack of toxicity towards erythrocytes, guavanins 2, 12, 13, and 14 presented potential for drug development. It is important to highlight that the determined MICs did not directly correlate with the calculated fitness values (as well as none of the other properties, such as charge or hydrophobicity). As shown in Supplementary Table 3, guavanins 1, 2, and 3 showed fitness values of 0.393, 0.390, and 0.390, respectively; however, their MICs are 200, 6.25, and > 200 μg mL^−1^, respectively (Supplementary Table 3). Therefore, while the fitness function employed here successfully created AMP sequences, it was unable to systematically predict the MIC of guavanin sequences. Another important point is that the parent peptides, the Pg-AMP1 fragments, were not active against *P. aeruginosa* under the same criteria; however, the charge fragment was toxic towards human erythrocytes at 100 μg mL^−1^ (Supplementary Table 3). This clearly shows that despite suboptimal solutions, more than half of the guavanins had their antimicrobial activity improved and toxicity reduced.

### Guavanin 2 has a narrow spectrum of activity

Because guavanin 2 was the most potent peptide identified in the screening step (Supplementary Table 3), it was selected for in depth analysis, using purified peptides synthesized by solid phase. Guavanin 2 was highly active against Gram-negative bacteria, particularly *Escherichia coli* and *Acinetobacter baumannii*, and exhibited only limited activity towards *Klebsiella pneumoniae* (Table 1). Conversely, the peptide showed very modest killing activity towards Gram-positive bacteria (Table 1). The antifungal profile of guavanin 2 was also modest, exhibiting poor killing of the yeast *Candida parapsilosis* and was inactive against *Candida albicans* (Table 1). The antibiofilm activity was also tested against *E. coli, Staphylococcus aureus, K. pneumoniae*, and *C. albicans*, showing that guavanin 2 was only able to reduce *C. albicans* biofilms (Supplementary Fig. 2).

**Table 1:** Antimicrobial activity and cytotoxicity of synthetic peptide guavanin 2

Cell strain	Microorganism/cell Line	Active concentration (µM)^a^
Gram-negative bacteria	*Escherichia coli* ATCC 25922	6.25
	*Pseudomonas aeruginosa* ATCC 27853^b^	25
	*Klebsiella pneumoniae* ATCC 13883	80
	*Acinetobacter baumannii* ATCC 19606	6.25
Gram-positive bacteria	*Staphylococcus aureus* ATCC 25923	100
	*Streptococcus pyogenes* ATCC 19615	50
	*Listeria ivanovii* Li4pVS2	50
	*Enterococcus faecalis* ATCC 29212	>100
Yeast	*Candida albicans* ATCC 90028	>200
	*Candida parapsilosis* ATCC 22019	≥50
Human cells	Erythrocytes	>200
	HEK-293 cells	>200

^a^ The minimum inhibitory concentrations (MIC) for microorganisms, the lytic concentration 50 (LC_50_) for erythrocytes, and the inhibitory concentration 50 (IC_50_) for HEK-293 cells are expressed as average values from three independent experiments performed in triplicate

^b^ MIC against *P. aeruginosa* differs due to experimental conditions (see Methods)

### Guavanin 2 kills bacteria with slow membranolytic kinetics

**Fig. 2:**
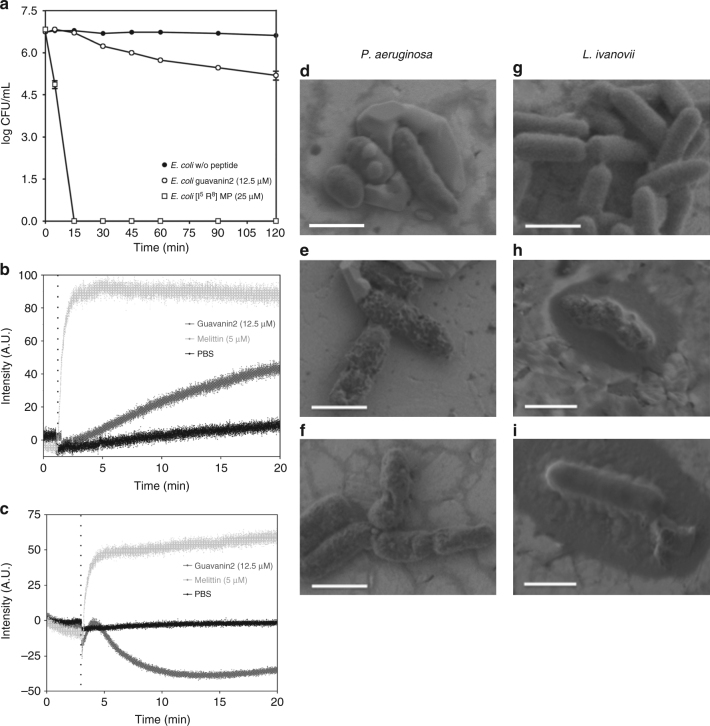
Killing and membrane effects of guavanin 2. **a** Time–kill profile of guavanin 2 against *E. coli* ATCC25922. Positive and negative controls correspond to bacteria incubated [I^5^, R^8^] mastoparan and without peptide, respectively. The data are the means ± S.E.M. of one experiments executed in triplicate. **b**, **c** Effect of guavanin 2 on plasma membrane integrity of *E. coli* ATCC 25922 cells after addition (vertical dotted line) of a concentration of peptide twofold above the MIC (12.5 µM). The pore-forming peptide melittin (5 µM) was used as a positive control. The negative control PBS corresponds to the bacteria incubated with the fluorescent probes without peptide. **b** Time-course cytoplasmic membrane permeation analysis of SYTOX Green uptake. **c**Cytoplasmic membrane hyperpolarization using DiSC3(5). **d** SEM-FEG visualization of the effect of guavanin 2 on *P. aeruginosa* (**d**–**f**) and *L. ivanovii* (**g**–**i**). The Controls without peptide are displayed in the **d**, **g** panels, respectively.  Bacteria were treated with a concentration of guavanin 2 corresponding to 25 µM (**e**), 50 µM (**f**, **h**) and 100 µM (**i**), respectively. Scale bar = 1 µm

The killing kinetics of guavanin 2 against *E. coli* revealed that after 120 min of incubation at a peptide concentration of 12.5 µM (twofold above the MIC), approximately 20% of *E. coli* cells were killed (a reduction from 10^7^ to ~10^5^ colony-forming units), in contrast to our recently developed [I^5^, R^8^] mastoparan peptide^24^ that completely killed *E. coli* within 15 min (Fig. 2a). As the main target of many AMPs is the bacterial membrane, we analyzed the membrane permeability and depolarization of *E. coli* cells with SYTOX Green (SG) and DiSC3(5), respectively, with a peptide concentration identical to that used in the time–kill assays. As shown in Fig. 2b, a rapid and maximal SG fluorescence signal was reached after incubation of bacteria with 5 µM of melittin, a 26-residue AMP from bee venom that acts on bacterial membranes via pore formation^25^ and serves as a positive control for membrane damage. In contrast, guavanin 2 caused only a slow and very small amount of dye influx in comparison to the positive and negative controls. Surprisingly, we observed a decrease in DiSC3(5) fluorescence after incubating *E. coli* cells with guavanin 2 (Fig. 2c), suggesting that this peptide induces hyperpolarization of the bacterial membrane, unlike melittin that produced a rapid increase in the fluorescence signal. Thus, guavanin 2, unlike most other AMPs, acts by hyperpolarizing the bacterial membrane. In order to obtain more insights into the killing mechanism of guavanin 2, we performed a complementary scanning electron microscopy with field emission gun (SEM-FEG) analysis of the sensitive Gram-negative bacteria, *P. aeruginosa* ATCC 27853, and the Gram-positive bacterium, *L. ivanovii* Li4pVS2. SEM-FEG images clearly show membrane damage (deformations/indentations) of *P. aeruginosa* cells after incubation with 25 µM (MIC) and 50 µM of guavanin 2 in comparison to intact bacteria (Fig. 2d). Similar results were observed for *L. ivanovii* (Fig. 2d).

### Guavanin 2 has a safe in vitro selectivity index

In drug development, it is important that a drug candidate presents a safe therapeutic profile wherein the amount of drug required to achieve a therapeutic effect is significantly lower than the amount that causes toxicity towards human cells. Here, we evaluated the in vitro selectivity index of guavanin 2, which is analogous to the therapeutic index. Guavanin 2 toxicity was investigated toward human erythrocytes and embryonic kidney cells (HEK-293). No hemolytic activity (LC_50_ higher than 200 µM) or cytotoxicity towards HEK-293 cells (IC_50_ higher than 200 µM) were observed (Table 1). Taking into account the MICs against Gram-negative bacteria and the cytotoxicity assessments, guavanin 2 showed a selectivity index of 23.93, indicating that to achieve a toxic effect a 24-fold administration of this peptide would be necessary, in contrast to Pg-AMP1, which displays a selectivity index of 4.88 (based on data from Tavares et al.^26^).

### Guavanin 2 anti-infective activity in a murine model

**Fig. 3:**
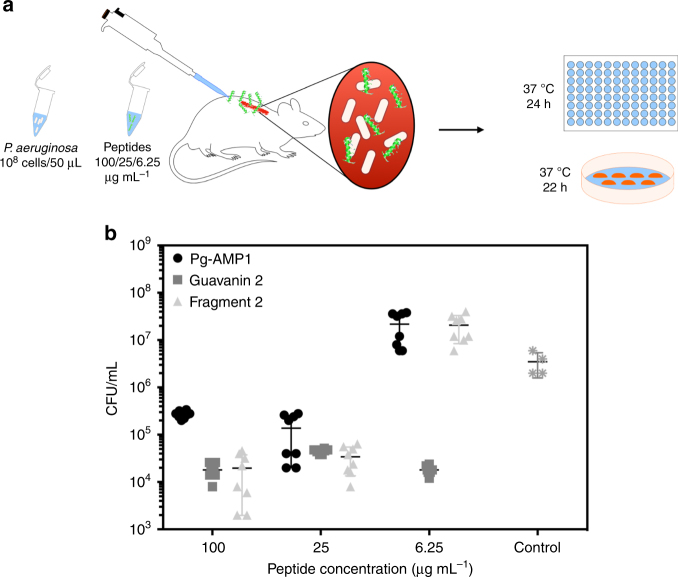
In vivo activity of guavanin 2. **a** Schematic of the experimental design. Briefly, the back of mice was shaved and an abrasion was generated to damage the stratum corneum and the upper layer of the epidermis. Subsequently, an aliquot of 50 μL containing 5 × 10^7^ CFU of *P. aeruginosa* in PBS was inoculated over each defined area. One day after the infection, peptides Pg-AMP1, guavanin 2, and Pg-AMP1 charge fragment were administered to the infected area. Animals were euthanized and the area of scarified skin was excised. **b** Four  days post-infection, homogenized using a bead beater for 20 min (25 Hz), and serially diluted for CFU quantification. Two independent experiments were performed with four mice per group in each case. Statistical significance was assessed using a two-way ANOVA. At all doses tested treatment with guavanin 2 significantly reduced CFU counts (*p* < 0.0001). Treatment with Pg-AMP1 and fragment 2 led to a significant reduction of bacterial load only at higher concentrations (25 and 100 µg mL^−1^)

In order to compare the anti-infective potential of guavanin 2 with respect to its ancestors (Pg-AMP1 and fragment 2), we leveraged an established *P. aeruginosa* abscess skin infection mice model, since in this infection model, the toxicity towards erythrocytes of guavanin 2's ancestors is irrelevant^27,28^, and the three peptides have some activity against this bacteria, which could cause skin infections in healthy or immunocompromised patients^29^. Therefore, mice were infected and peptides were administered to the site of infection 24 h later (Fig. 3). Treatment with guavanin 2 led to a 3-log reduction in bacterial counts after 4 days, even at the lowest dose tested of 6.25 μg mL^−1^ (Fig. 3). On the other hand, naturally occurring wild-type peptide Pg-AMP1 exhibited no activity at 6.25 μg mL^−1^ (Fig. 3). All peptides displayed comparable anti-infective activity at higher concentrations (25 and 100 μg mL^−1^).

### Guavanin 2 structure depends on environment hydrophobicity

**Fig. 4:**
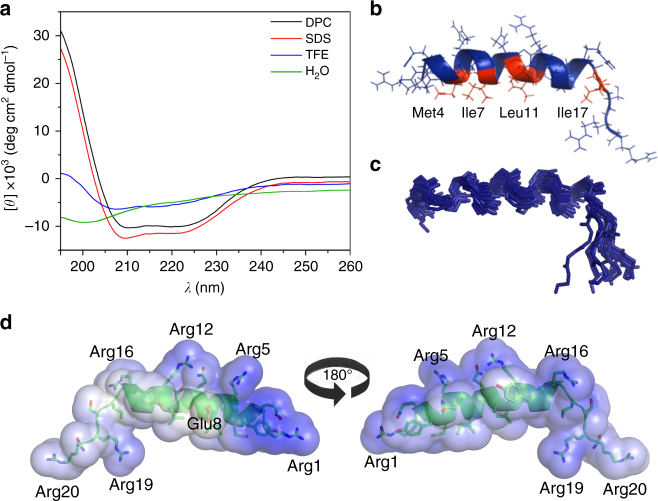
Structure analysis of guavanin 2. **a** CD spectra of guavanin 2. The spectra in DPC (20 mM), SDS (20 mM) and TFE 50% were obtained at 25°C, pH 4 and a peptide concentration of 38 μM. The spectra in water was obtained at 25°C, pH 7 and a peptide concentration of 33 μM. Guavanin 2 has no defined structure in water, whereas it presents α-helical structure with hydrophobic solvents, in special DPC, which was selected for solving the three-dimensional structure by nuclear magnetic resonance (NMR). **b** Solution NMR structure of guavanin 2 in 100 mM (DPC-d_38_) micelles; a ribbon representation structure of lowest energy structure with side chains labeled (blue is hydrophilic and red is hydrophobic). **c** Ensemble of 15 backbone structures with low energy. **d** Electrostatic surfaces of guavanin 2 in 100 mM (DPC-d_38_) micelles. Surface potentials were set to ±5 kT e^−1^ (133.56 mV). Blue indicates positively charged regions and white apolar ones. Charged residues are labeled

According to ab initio molecular modeling, guavanins 1–15 showed an  α-helical structure (Supplementary Fig. 1). To confirm these data, guavanin 2 was used as the prototype for in vitro structural analysis. As the target of guavanin 2 is the bacterial membrane (Fig. 2), we performed a structural analysis to verify whether there was a conformational change of guavanin 2 in hydrophobic environments, and also evaluate whether the fitness function of the genetic algorithm was able to generate a peptide capable of adopting an α-helical structure. Circular dichroism (CD) experiments of guavanin 2 were performed in water (pH 7) indicating no defined secondary structure (Fig. 4a). At the same pH, an α-helical conformation was observed in sodium dodecyl sulfate (SDS) micelles (Supplementary Fig. 3), indicating a coil-to-helix transition upon interaction with hydrophobic environments. The pH influence on the structure was also tested in SDS micelles, showing that guavanin 2 maintained the α-helical structure at pH 4, 7, and 10, where in pH 4 it showed the higher abundance of secondary structure (Supplementary Fig. 3). To determine the best environment for NMR experiments, guavanin 2 was tested in SDS, dodecyl-phosphocoline (DPC), and 2,2,2-Trifluoroethanol (TFE). In SDS and DPC micelles (20 mM) at pH 4, the peptide showed the highest abundance of secondary structure, presenting 42% and 39% of α-helical content, respectively (Fig. 4a).

The three-dimensional structure of synthetic guavanin 2 in the presence of deuterated dodecyl-phosphocoline (DPC-d_38_) micelles, which are routinely used as a membrane-mimetic^30,31^, was elucidated using 2D nuclear magnetic resonance (NMR) spectroscopy and the structural statistics for low-energy structures are summarized in Table 2. ^1^H-^1^H NOESY spectra revealed a total of 358 distance restraints with 17.9 average restrictions per residue.

**Table 2:** NMR and refinement statistics for peptide structures

	Protein
NMR distance and dihedral constraints	
Distance constraints	
Total NOE	358
Intra-residue	204
Inter-residue	
Sequential (|*i* – *j*| = 1)	116
Medium-range (|*i* – *j*| < 4)	38
Long-range (|*i* – *j*| > 5)	0
Intermolecular	
Hydrogen bonds	0
Total dihedral angle restraints	
*ϕ*	16
*ψ*	16
**Structure statistics**	
Violations (mean and S.D.)	
Distance constraints (Å)	0.05 ± 0.05
Dihedral angle constraints (°)	9.21 ± 8.38
Max. dihedral angle violation (°)	35.35
Max. distance constraint violation (Å)	0.26
Deviations from idealized geometry	
Bond lengths (Å)	0.0181 ± 0.0001
Bond angles (°)	0.54 ± 0.01
Impropers (°)	0.47 ± 0.04
Average pairwise r.m.s. deviation^a^ (Å)	
Heavy	2.28 ± 0.33
Backbone	1.37 ± 0.34

^a^Pairwise r.m.s. deviation was calculated among 10 refined structures for residues 1–20

Guavanin 2 adopted an α-helical structure between residues Gln^2^–Arg^16^ in 100 mM of DPC-d_38_ micelles, supporting the ab initio predictions (Supplementary Fig. 1). The 10 structures are highly precise, with a backbone RMSD of 0.88 ± 0.25 Å (mean + S.D. of nine samples) over residues 2–16. Despite the random character of C-terminal region, the heavy atoms RMSD equivalent to 2.28 ± 0.33 (mean + S.D. of nine samples), revealed that the 10 structures were well defined and concise in DPC-d_38_ micelles. Intra-side-chain interactions also contributed to the good geometry of the peptide. The residues Arg^1^, Gln^2^ and Tyr^3^ are involved in hydrogen bonding network that stabilizes the N-terminal region; while Gln^9^ interacts with Arg^5^ or Arg^12^, stabilizing the center of the structure (Supplementary Fig. 4). Guavanin 2 forms a relatively well ordered apolar cluster with aliphatic residues Met^4^, Ile^7^, Leu^11^, and Ile^17^ (Fig. 4b). Thus, the existence of converging conformations showed regularity and agreement among the restraints used in the structural calculation (Fig. 4c). The electrostatic potential on the surface of the peptide structure revealed that guavanin 2 is highly cationic, suppressing the negative charge of Glu^8^ (Fig. 4d) and generated a solvation potential energy of 2.38 ± 0.33 MJ mol^−1^ (mean + S.D. of 10 samples). Depending on the N-Terminal protonation, the net charge of guavanin 2 varies from +5 to +6, as the C-Terminal is amidated. This net charge likely promotes the attraction of guavanin 2 to cell membranes composed of phospholipids with negatively charged head groups, which is considered the first stage of its mechanism of action towards Gram-negative cells.

## Discussion

The first plant AMPs were identified in the 1970s and since that time a range of different classes of AMPs have been identified^5^. Overall, they are composed  of tens of amino acids residues, have an uncommon composition and structures stabilized by disulfide bridges. The complexity of their chemical structures is perhaps the main disadvantage of plant based AMPs and likely contributes to why none have reached the market^5^. Promising design methods have recently been applied to engineer AMPs and help overcome such limitations while simultaneously increasing AMP potency and reducing cytotoxicity towards human cells^10^. Unfortunately, many of these methods are based on incremental modifications of an AMP template and when peptides are designed from scratch they often share similarity with AMP sequences found in databases and consequently only a very limited set of amino acids are harnessed to design the synthetic AMPs.

The present study describes the application of computer-aided design techniques to explore the unique composition of plant AMPs to engineer innovative synthetic AMPs. We leveraged a custom genetic algorithm to optimize the guava peptide, Pg-AMP1, and generated the guavanin peptides one of which displayed potent activity against Gram-negative bacteria. The genetic algorithms were previously applied in the field of AMP design^15,17,18^. However, our custom genetic algorithm presents two main modifications for designing truly innovative peptides: first, the application of an equation instead of a machine learning classifier, and second the interruption of the algorithm before it reaches a plateau.

The fitness function was implemented as an equation that relates hydrophobic moment and α-helix propensity (see Methods), thus guiding the algorithm to select for amphipathic and α-helical peptides but not necessarily sequences that correspond to traditional AMPs. Due to the improvement in the hydrophobic moment, two kinds of amino acids would be preferentially selected during the iteration steps: both positively charged (mainly Arg residues) and hydrophobic residues (Leu and Ile residues). Therefore, the application of the fitness function should favor a peptide with a segregation of positively charged and hydrophobic residues that adopts an α-helical structure, which is a well know property of many conventional AMPs^6,7,10^.

After hundreds of algorithm iterations, an optimal solution for this type of mathematical modeling would result in peptides composed primarily of Ala, Arg, Ile, and Lys residues (as observed by Patel et al.^17^ and Maccari et al.^15^). However, in order to obtain peptides with uncommon composition that do not exist in nature, the algorithm was set to promote slow optimization (200 from 250 sequences were promoted for the next iteration and only 0.5% of mutations were allowed) and the iterations were stopped at the 50th one, before the fitness function plateaued and with no hits to CAMP sequences (Fig. 1c). Therefore, we reached a suboptimal solution for our mathematical model and our designed AMPs exhibited unique amino acid composition compared to sequences found in the APD (Fig. 1d).

**Fig. 5:**
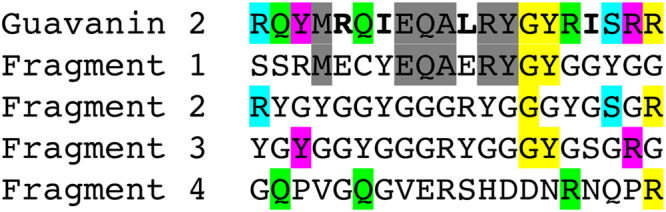
Guavanin 2 and its ancestors. Guavanin 2 and the Pg-AMP1 fragments were aligned without including gaps to demonstrate the guavanin 2 inherited residues and mutations. The residues inherited from each the fragments are highlighted in gray (fragment 1—α-helix propensity), cyan (fragment 2—net charge), magenta (fragment 3—hydrophobicity), light green (fragment 4—hydrophobic moment), and yellow (two or more fragments); and the mutated residues are in bold face

The computationally designed guavanins were found to be rich in arginine residues (and some of them are also tyrosine-rich), whereas the parent peptide, Pg-AMP1, is classified as a glycine-rich peptide; it is important to remember that four Pg-AMP1 fragments were used in the founder population (Fig. 1a,b) and three of them were rich in tyrosine residues (Fig. 5). During the algorithm iterations, Gly residues tended to disappear, as they do not favor α-helix formation (Fig. 5). Conversely, Arg residues were rapidly fixed in the derived populations, as this residue serves as the cationic counterpart of the peptide and has a good α-helix propensity (Fig. 5). As the algorithm promoted slow optimization, Tyr residues were retained as the hydrophobic counterpart of the peptide; however, there would be a tendency to replace them by Leu or Ile residues with more iteration steps (Fig. 5). Ultimately, owing to the slow optimization process, the most active peptide, guavanin 2, possessed a residual Gly residue and only four accumulated mutations (Fig. 5). In this regard, it is important to highlight that genetic algorithms are highly sensitive to operators and parameters choice. Since our fitness function is dependent on physicochemical scales, the outcome could vary according to the scale used. Here we used Eisenberg’s hydrophobicity scale^32^, which has an amplitude of 2.53, whereas the Kyte–Doolittle^33^ and Radzicka–Wolfenden^34^ scales have amplitudes of 9.00 and 19.84, respectively. The application of the Kyte–Doolittle and Radzicka–Wolfenden scales changes the search landscape, because their amplitudes favor the hydrophobic moment in relation to the helix propensity.

This approach was capable of designing AMPs, as 8 out of 15 guavanins (53%) were categorized as AMPs, following our criteria of classification of peptides as antimicrobial (Supplementary Table 3) and all of these had lower MICs than the four Pg-AMP1 fragments used as starting peptide sequences (Supplementary Table 3). In addition, all the modeled guavanins were predicted to form an α-helix as the secondary structure (Supplementary Fig. 1 and Supplementary Table 2). It is important to highlight that the fitness function was developed to select amphipathic α-helices, not necessarily conventional AMPs, as the hydrophobic moment is not correlated with MIC values^35^. Its accuracy for AMPs (53%) is reasonable, because none of the currently available antimicrobial activity prediction tools have accuracies higher than 50% for synthetic peptides^16^. However, the accuracy of the fitness function for selecting AMPs may be improved by increasing the number of iterations (which would retrieve sequences similar to those of known AMPs as depicted in Fig. 1c) and/or by the addition of structural parameters such as solvation potential energy^36^, although this would consume more time and computational power because it requires three-dimensional structure prediction.

Structural studies performed using CD and NMR spectroscopy demonstrated that our approach successfully generated an α-helical peptide. The CD studies indicated that guavanin 2 was unstructured in aqueous solution, but formed a well-defined structure composed of an α-helix in the presence of micelles or structure-inducing solvents (Fig. 4a). The NMR analysis revealed that guavanin 2 formed an α-helical structure between residues Gln^2^–Arg^16^ in the presence of 100 mM DPC-d_38_ micelles, further supporting the CD structural data and suggests that guavanin 2 adopts a predominantly α-helical conformation in the presence of a biological membrane.

Further characterization of the biological properties of guavanin 2 revealed that this peptide acted preferentially against Gram-negative bacteria (Table 1) with a selectivity index of 23.93. As this index is analogous to the therapeutic index, guavanin 2 could be considered a safe peptide, as according the U.S. Food and Drug Administration, a therapeutic index is considered narrow when it is below two, while for a safer drug, the higher the index, the better the drug^37^. Because Pg-AMP1 is hemolytic^26^, as well as its second fragment (Supplementary Table 3), they are not appropriate for intravenous use; therefore, in order to compare them in an animal model, we used a wound infection  mouse model, where the toxic effects, in comparison with systemic administration, are reduced. This experiment indicated that, pharmacologically, guavanin 2 was better than its ancestors (Pg-AMP1 and Pg-AMP1 fragment 2) because guavanin 2 kept its activity after 4 days at a dose of 6.25 μg mL^−1^, whereas the ancestors needed a dose of 25 μg mL^−1^. Previously, our group demonstrated the effects of cycloviolacin O2 and kalata B2 against *S. aureus* in a similar in vivo model^28^, showing similar results from guavanin 2 against *P. aeruginosa*. Despite the similar activity, guavanin 2 is easier to obtain by chemical synthesis, making it better than cyclotides, which need several post-translational modifications to reach the active form^38^.

Since guavanin 2 is an AMP, we investigated its mechanism of action. We demonstrated that this peptide kills *E. coli* cells in a relatively slow manner (Fig. 2a), similarly to temporin-SHd^39^. In addition, SEM-FEG imaging indicated that guavanin 2 induces bacterial membrane damage (Fig. 2d). It is important to highlight that the membranolytic activity of guavanin 2 is different from melittin and our recently designed peptide [I^5^, R^8^] mastoparan^24^. In this case, the killing was eightfold slower than that of [I^5^, R^8^] mastoparan and it was observed that guavanin 2 also slowly permeated the cytoplasmic membrane by inducing membrane hyperpolarization, in contrast to melittin (Fig. 2c). In fact, the peptide-induced hyperpolarization indicates that guavanin 2 could act as a selective ionophore, similar to the antimicrobial compounds valinomycin^40^ and citral^41^, which are selective for potassium ions. Altogether, these results suggest that the potent effect of guavanin 2 observed against *E. coli* (MIC of 6.25 µM) is due to pore formation within the cytoplasmic membrane.

The hyperpolarization effect could be a reflection of guavanin 2 amino acid composition (30% of guavanin 2 is composed of arginine residues) and uncommon amino acids for AMPs (three tyrosine and three glutamine residues), which in turn shows that the inclusion of non-proteinogenic amino acids (e.g. norleucine, ornithine)^15,19^ is not necessarily essential to obtain innovative peptides. In fact, it is difficult to escape from the utilization of Arg or Lys residues, despite the fact that some AMPs use His residues^21^, as these are the only residues that possess positively charged side chains. In addition, we demonstrated that it is possible to use other hydrophobic residues, Tyr in this case, as the hydrophobic counterpart of the peptide.

In the present study, we have demonstrated that the in silico evolved and optimized peptide, guavanin 2, is a better candidate for drug development than the naturally occurring peptide Pg-AMP1. We have also demonstrated that natural plant peptides could have a critical role in identifying AMP sequences with therapeutic potential. The artificial peptide guavanin 2 has a different mechanism of action than most AMPs as it causes membrane hyperpolarization, whereas other peptides depolarize it. Manipulation of natural AMP sequences using the computational approach described here could lead to innovative combinations of amino acids and may constitute a platform for the development of designed AMPs with distinct mechanisms of action.

## Methods

### Genetic algorithm

The genetic algorithm simulates the evolution of a population of sequences during a number of iterations, where given iteration I_*n*_ generates the population P_*n*_ from the population P_*n*−1_, evaluating the sequences according to the value of a fitness function (see below: Eq. 1), also known as chance of survivor and mating (Fig. 1). The algorithm was implemented in PERL. In the first iteration (I_1_) of the implementation of our custom genetic algorithm, all sequences from P_0_ had the same fitness value, thus providing a random selection for each sequence pair (Fig. 1). From iteration I_2_ to I_*n*_, the sequence selection for mating was performed according to the corresponding fitness values by means of a roulette wheel selection model. For each iteration, 250 sequence pairs were selected from population P_*n*_ and each pair was submitted to a crossing over process, generating a couple of children for population P_*n*+1_. Each children had a 0.5% chance of mutation, where one residue was randomly selected for substitution. The replacement was chosen according to the probability distribution listed in Supplementary Table 4. From the replacing residues list, Gly and Pro were removed due to poor α-helix formation; Asp and Glu due to their negative charge; and Cys due to the possibility to form disulfide bridges. Next, the sequences from P_*n*+1_ were evaluated by the fitness function. The 50 worst sequences were removed from the population P_*n*+1_ and then another iteration step was initiated (Fig. 1b). The cycle was repeated until the number of iterations was exhausted. For the development of synthetic guavanins, 100 independent simulations were performed, each one with 400 iterations using the same conditions. The sequence with the highest fitness values, among all independent simulations, at iterations 50, 100, 200, and 400 was subjected to a BLAST search^42^ against the CAMP database^43^ in order to select the iteration that generated sequences which lacked similarity to known AMPs. Finally, the best sequence from each independent simulation at the 50th iteration was chosen and then the set was ranked; the 15 best sequences, according to the fitness function, were selected for further evaluation.

### Fitness function

The Eq. (1) was designed to generate amphipathic α-helical peptides, based on the ratio between Eisenberg’s hydrophobic moment and the sum of exponential α-helix propensity in Pace–Schols scale:

1 $${\mathrm{Fitness}} = \frac{{\root {2} \of {{\left[ {\mathop {\sum }\nolimits_{i = 1}^I H_i \times {\mathrm{cos}}(\delta i)} \right]^2 + \left[ {\mathop {\sum }\nolimits_{i = 1}^I H_i \times {\mathrm{sin}}(\delta i)} \right]^2}}}}{{\mathop {\sum }\nolimits_{i = 1}^I e^{{\mathrm{Hx}}_i}}},$$

where *δ* represents the angle between the amino acid side chains (100° for α-helix, on average); *i*, the residue number in the position *i* from the sequence; *H*_*i*_, the *i*th amino acid’s hydrophobicity on a hydrophobicity scale; Hx_*i*_, the *i*th amino acid’s helix propensity in Pace–Schols scale^44^; and *I*, the total number of residues present in the sequence.

Instead of directly using the hydrophobic moment equation, we introduced modifications into the equation to account for α-helix propensity, because we observed that in Pg-AMP1, the C-terminal portion showed the highest hydrophobic moment (Fig. 1a and S2), but in our previous studies this portion was intrinsically unstructured^26,45^. Therefore, the hydrophobic moment per se does not guarantee α-helix formation. As the Pace–Schols α-helix propensity is given in terms of the amount of energy required for a given amino acid residue to adopt an α-helical conformation (i.e. the lower energy, the easier for that residue to adopt an α-helical conformation), we introduced the α-helix propensity in the denominator of Eq. (1). However, using the α-helix propensity in the denominator has a bias: as the scale is normalized by subtracting the resulting values from that of alanine; thus, the normalized value of alanine is zero. Therefore, the algorithm tends to lower the value of α-helix propensity because it is in the denominator. However if α-helix propensity reaches a zero value, it would generate a division by zero (formally $\alpha {\mathrm{/}}0 = \infty$, being *α* a positive number), hindering the algorithm progress. Therefore, by using the exponential values of Pace-Schols scale, we could avoid the division by zero (as $e^0 = 1$).

### Computational selection of Pg-AMP1 fragments

In order to identify Pg-AMP1 (‘P86030 [http://www.uniprot.org/uniprot/P86030]’) regions with potential to have antimicrobial activity, the Pg-AMP1 sequence was submitted to a sliding window system, generating 36 fragments of 20 amino acid residues, which corresponds to the average AMP length^14^. For each fragment, four independent properties were calculated: α-helix propensity, positive net charge, hydrophobicity, and hydrophobic moment. For each property, one fragment was selected (Fig. 1). The α-helix propensity was calculated by using the α-helix propensity scale from Pace and Scholtz^44^ and the hydrophobicity and hydrophobic moment were measured using the Eisenberg’s hydrophobic scale^32^. The hydrophobic moment was calculated using the Eisenberg’s equation^32^. The composition of guavanins was compared with APD2 ^46^, for general and α-helix peptides; and PhytAMP^47^ for plant peptides.

### Ab initio molecular modeling

Three-dimensional models of the 4 Pg-AMP1 fragments and the 15 best fitness guavanins were generated by means of QUARK ab initio modeling server^48^. The models were assessed by means of ProSA II^49^, PROCHECK^50^ and MODELLER 9.17 ^51^. The stereochemical quality of a protein structure was assessed by the Ramachandran plot, which is generated by PROCHECK. Reliable models are expected to have more than 90% of amino acid residues in most favored and additional allowed regions. The G-factor, which is calculated by PROCHECK, is a measurement of how unusual the structure is, G-factor values below −0.5 are unusual. The fold quality is indicated by the *Z*-score of ProSA II. The MODELLER 9.17 ^51^ build in function for the discrete optimized protein energy score (DOPE score) was also to assess the energy of the models.

### High-throughput peptide synthesis on cellulose arrays

A peptide array composed of 20 peptides (15 guavanins, 4 Pg-AMP1 fragments, and magainin 2) was designed and synthesized by Kinexus Bioinformatics Corporation (Vancouver, BC). Peptides were produced in a standard mass of 80 μg by using cellulose support in SPOT technology^23^. The crude synthetic peptides were obtained from cellulose membrane discs that had already been treated with ammonia gas to release the peptides from the membrane. Peptides were then dissolved overnight in distilled water and subsequently evaluated for their biological activities, as described below.

### Bioluminescent determination of antimicrobial activity

The antimicrobial activity of the synthesized peptides was evaluated against an engineered luminescent *P. aeruginosa* H1001 (*fliC::lux*CDABE) strain in 96-well microplates^52^. Aqueous solutions of peptides released from the cellulose spots were diluted twofold in BM2 medium [62 mM potassium phosphate buffer pH 7; 2 mM MgSO_4_; 10 μM FeSO_4_; 0.4% (wt/vol) glucose] down the eight wells of a 96-well plate, achieving a final volume of 25 μL in each well. Subsequently, 50 μL of overnight culture of *P. aeruginosa* H1001 (*fliC::lux*CDABE) were subcultured in 5 mL of fresh LB media and grown until they reached an OD_600_ of 0.4. This growing bacteria culture was then diluted 4:100 (v/v) into fresh BM2 media and 25 μL of this diluted bacterial culture was transferred to the microplate wells containing 25 μL of peptide solution. The final peptide concentrations tested ranged from 200 to 3 μg mL^−1^. The plates were incubated for 4 h at 37 °C with constant shaking at 50 rpm. Luminescence was measured on a Tecan SPECTRAFluor Plus Microplate Reader (Tecan US, Morrisville, NC). The antimicrobial activity was evaluated by the ability of the peptides to reduce the luminescence of *P. aeruginosa*-lux strain compared to untreated cells. The AMP magainin 2 and the carbapenem meropenem were used as positive controls and distilled water was used as a negative control.

### Hemolytic assays

Fresh human venous blood was collected from volunteers in Vacutainer collection tubes containing sodium heparin as an anticoagulant (BD Biosciences, Franklin Lakes, NJ). The blood was centrifuged applying 151 × *g* force (1500 rpm) and the serum was removed and the blood cells were replaced and washed three times with the same volume of sterile NaCl 0.85% solution. Concentrated red blood cells were diluted tenfold in NaCl 0.85% solution and then exposed at twofold dilutions of peptides for 1 h at 37 °C, at identical concentrations used for antimicrobial assays, in the ratio of 1:1 (v/v), achieving a final volume of 100 μL. The assay was carried out in 96-well polypropylene microtiter plates. The positive control wells contained 1% of Triton X-100, representing 100% cell lysis, and negative control wells contained sterile saline. Hemoglobin release was monitored chromogenically at 546 nm using a microplate reader. The experiments were approved by Clinical Research Ethics Board from University of British Columbia (CA), certificate number H04-70232, and all blood donors consent in participate voluntarily in the experiments.

### Peptide synthesis by solid phase

The peptide guavanin 2 was synthesized by stepwise solid phase using the *N*-9-fluorenylmethyloxycarbonyl (FMOC) strategy and purified by high-performance liquid chromatography, with purity >95% by Peptide 2.0 (Virginia, USA). The sequence and degree of purity (>95%) was confirmed by MALDI-ToF analyses^13^.

### Antimicrobial activity

The antimicrobial activity spectrum of guavanin 2 was determined by growing the microorganisms in the presence of twofold serial dilutions of the peptide in 96-well microtitre plates^53^. Lysogeny Broth (LB) was used for cultures of *Acinetobacter baumannii* ATCC 19606, *Enterococcus faecalis* ATCC 29212, *Escherichia coli* ATCC 25922, *Klebsiella pneumoniae* ATCC 13883, *Pseudomonas aeruginosa* ATCC 27853, and *S. aureus* ATCC 25923. Brain Heart Infusion (BHI) broth was used for cultures of *Listeria ivanovii* Li 4pVS2 and *Streptococcus pyogenes* ATCC 19615. Yeast peptone dextrose (YPD) medium was used for *Candida* species (*C. albicans* ATCC 90028 and *C. parapsilosis* ATCC 22019). MH (Mueller Hinton) broth was used for suspend centrifuged culture of bacteria and yeasts in logarithmic phase to an *A*_630_ of 0.01 (~10^6^ cfu mL^−1^), except for *S. pyogenes*, *L. ivanovii*, and *E. faecalis* that were suspended in their respective growth medium. Guavanin 2 serial dilutions with final concentrations ranging from 200 to 1 µM in 50 µL were mixed with 50 µL of the microorganism suspensions and incubated during 18 h at 37 °C (30 °C for yeasts). A microplate reader (UVM 340, Asys Hitech) was used to monitor the antimicrobial susceptibility by measuring the change in *A*_630_. The minimal inhibitory concentration (MIC) was determined as the lowest peptide concentration that completely inhibited the growth of the microorganism and corresponds to the average value obtained from three independent experiments. Each experiment was performed in triplicate with positive (0.7% formaldehyde) and negative (without peptide) inhibition controls.

### Antibiofilm assays

Overnight cultures of microorganisms [*E. coli* 0157, methicillin-resistant *S. aureus* (MRSA), *K*. *pneumoniae* 1825971, or *C*. *albicans* clinical isolate obtained from a blood culture maintained at LACEN (Laboratório Central de Saúde do Distrito Federal, a Brazilian reference laboratory for clinical isolates in Brasília, Brazil)] grown in LB broth (BD Difco) were diluted 1 in 100 in LB and Todd-Hewitt broth (BD Difco) or BM2 minimal medium [62 mM potassium phosphate buffer, pH 7.0, 7 mM (NH_4_)_2_SO_4_, 2 mM MgSO_4_, 10 μM FeSO_4_, 0.5% glucose], and 100 μL per well was added into 96-well round-bottom microplates. Absorbance at 600 nm was used to assess the planktonic cell growth. Non-adherent planktonic cells were removed from microtiter wells and washed twice with deionized water. Adherent cells were stained with 0.1% crystal violet for 20 min. The microplate wells were washed twice with deionized water, air dried, and solubilized with 110 μL of 70% ethanol. After establishing the best conditions for biofilm growth, guavanin 2 was evaluated for its ability to inhibit biofilm formation. Biofilm formation was assessed by measuring the absorbance at 600 nm using a microplate reader (Bio-Tek Instruments, Inc., USA). The microdilution method for cationic peptides was used to determine the MIC for planktonic cells. Three biological replicates were performed for each experiment involving eight technical replicates. An one-sided Student’s *t*-test was applied for verifying the effects on planktonic and biofilm cells with their respective controls with a critical value of 0.05, with the null hypothesis that guavanin 2 does not affect planktonic or biofilm cells.

### Pre-formed biofilm eradication

The pre-formed biofilm eradication of *C. albicans* was determined by flow cell experiments^54^. Flow cell chambers with channel dimensions of 1 × 4 × 40 mm were inoculated by injecting 400 µL of an overnight culture diluted to an OD_600_ of 0.05. To enable initial adherence, chambers were left without flow for 2 h after inoculation. Then, the medium (with or without sub-inhibitory concentrations of guavanin 2) was pumped through the system at a constant rate of 2.4 mL h^−1^ during 72 h at 37 °C. After this period, in order to limit the amount of planktonic and loosely attached cells within the flow cell chamber, the flow rate (90 rpm) was increased. All media used in flow cell assays supported the planktonic growth of *C. albicans*, as determined by growth curves. Biofilm cells were stained using Syto-9 prior to microscopy experiments. Microscopy was done using a confocal laser scanning microscope (Olympus, Fluoview FV1000). Imaris software package (Bitplane AG) was used for three-dimensional reconstructions and quantifications of the overall biofilm volume (µm^3^) and the percentage of live and dead cell volume. Experiments were performed at in triplicate.

### Cytotoxic profiles

We determined the cytotoxicity of guavanin 2 against the human embryonic kidney cell line HEK-293. HEK-293 cells were cultured in DMEM medium, and incubated at 37 °C in a humidified atmosphere of 5% CO_2_. Cell viability was quantified after peptide incubation using a methylthiazolyldiphenyl-tetrazolium bromide (MTT)-based microassay^55^. Briefly, cells were seeded on 96-well culture plates at a density of 5 × 10^5^ cells mL^−1^ and incubated 72 h at 37 °C with 100 µL of guavanin 2 at different concentrations (12.5–200 µM, final concentrations). Then, 10 µL of MTT (5 mg mL^−1^ in PBS) was added to each well and the cells were further incubated for 4 h in the dark. The formazan crystals formed by mitochondrial reductases in intact cells are insoluble in aqueous solutions and precipitate. Formazan crystals were dissolved using a solubilization solution (40% dimethylformamide in 2% glacial acetic acid, 16% sodium dodecyl sulfate, pH 4.7) followed by 1 h incubation at 37 °C under shaking (150 rpm). Finally, the absorbance of the resuspended formazan was measured at 570 nm. Data were analyzed with GraphPad Prism® 5.0 software to determine the inhibitory concentration 50 (IC_50_), which corresponds to the peptide concentration producing 50% cell death. Results were expressed as the mean of three independent experiments performed in triplicate.

### In vitro selectivity index calculation

The in vitro selectivity index is analogous to the therapeutic index concept, corresponding to the ratio between cytotoxic effect and antibacterial effect. The selectivity index of guavanin 2 was calculated according Chen et al.^56^ with minor modifications, using Eq. ( 2):

2 $${\mathrm{SI}} = \frac{{\root {n} \of {{\mathop {\prod }\nolimits_{i = 1}^n {\mathrm{Cytotoxic}}_i}}}}{{\root {m} \of {{\mathop {\prod }\nolimits_{j = 1}^m {\mathrm{Antibacterial}}_j}}}},$$

where *n* is the number of cytotoxic assays with different cells and *m* is the number of antimicrobial assays with different bacteria. For values higher than the maximum concentration tested, it was assumed twofold the maximum tested value (e.g. if the value is higher than 100, it was considered as 200)^56^.

### Time–kill studies

The killing kinetics of guavanin 2 against the Gram-negative bacterium *E. coli* ATCC 25922 was investigated^39^. Exponentially growing bacteria in LB were harvested by centrifugation, washed three times in PBS and suspended in the same buffer to a final concentration of 10^7^ cfu mL^−1^. One hundred microliters of this bacterial suspension was incubated with a dose of peptide corresponding to twofold the MIC. Then, aliquots of 10 µL were withdrawn at different times, diluted in LB, and spread onto LB agar plates. The cfu were counted after overnight incubation at 37 °C. Two experiments were carried out in triplicate and negative controls were run without peptide and positive controls with [I^5^, R^8^] mastoparan peptide^24^.

### SYTOX green uptake assay

Permeabilization of *E. coli* ATCC 25922 cytoplasmic plasma membrane induced by guavanin 2 was determined by fluorometric measurement of SYTOX green (SG) influx^57^. SG is impermeant to live cells and when the cell membrane is damaged, this dye penetrates into the cell and binds to intracellular DNA, due to its high-affinity to nucleic acids, leading to an increase in fluorescence. Exponentially growing bacteria (6 × 10^5^ cfu mL^−1^) were resuspended in PBS after centrifugation (1000 × *g*, 10 min, 4 °C) and washing steps. About 792 μL of the bacterial suspension was pre-incubated with 8 μL of 100 μM SG during 30 min at 37 °C in the dark. After addition of 200 μL of guavanin 2 (final concentration twofold above the MIC), with a final volume of 1 mL, the fluorescence was monitored for 1 h at 37 °C using a Varian Cary Eclipse fluorescence spectrophotometer, with excitation and emission wavelengths of 485 and 520 nm, respectively. Results correspond to a representative experiment with negative (PBS) and positive (melittin) controls. Three independent experiments were performed.

### Membrane polarization assay

To study the ability of guavanin 2 to alter the plasma membrane potential, we evaluated the membrane depolarization *E. coli* (ATCC 25922) using the membrane potential-sensitive fluorescent probe DiSC3(5) (3,3′-dipropylthiadicarbocyanine iodide)^58^. When the cytoplasmic membrane is intact, the fluorescent probe DiSC3(5) accumulates into the cytoplasmic membrane and then aggregates, causing self-quenching of the fluorescence. In the presence of a membrane-depolarizing agent, DiSC3(5) is released into the medium, leading to an increase in fluorescence that can be monitored over time^59^. Briefly, exponentially growing bacteria were centrifuged (1000 × *g*, 10 min, 4 °C), washed with PBS, and resuspended in the same buffer to an *A*_630_ of 0.1; then 700 µL of bacteria were pre-incubated with 1 µM DiSC3(5) in the dark during 10 min at 37 °C, and then 100 µL of 1 mM KCl were added in order to equilibrate the cytoplasmic and external K^+^ concentrations. After addition of guavanin 2 (200 µL, final concentration: twofold above the MIC), the changes in fluorescence were recorded at 37 °C for 20 min at an excitation wavelength of 622 nm and an emission wavelength of 670 nm (Varian Cary Eclipse fluorescence spectrophotometer). Three independent experiments were performed and results correspond to a representative experiment with negative (PBS) and positive (melittin) controls.

### SEM-FEG imaging

 SEM-FEG was used to obtain high-resolution images of the effect of guavanin 2 on the Gram-negative bacteria *P. aeruginosa* (ATCC 27853) and the Gram-positive *L. ivanovii* (Li 4pVS2). Bacteria in mid-logarithmic phase were collected by centrifugation (100 × *g*, 10 min, 4 °C), washed twice with PBS, and suspended in the same buffer at a density of 2 × 10^7^ cfu mL^−1^. Two hundred microliters of the bacterial suspension were incubated 1 h at 37 °C with the peptide guavanin 2 at a final concentration corresponding to the MIC and twofold above the MIC. As a negative control, cells were incubated in buffer without peptide. Microbial cells were then fixed with 2.5% glutaraldehyde, homogenized by gently inverting the tubes, and stored at 4 °C prior to SEM-FEG analysis. A Hitachi SU-70 Field Emission Gun Scanning Electron Microscope was used to record SEM-FEG images. The samples (gold plates where 20 µL of inoculum were deposited and dried under nitrogen) were fixed on an alumina SEM support with a carbon adhesive tape and were observed without metallization. In Lens Secondary electron detector (SE-Lower) was used to characterize our samples. The accelerating voltage was 1 kV and the working distance was around 15 mm. At least five to ten different locations were analyzed on each surface, leading to the observation of a minimum of 100 single cells.

### Scarification skin infection mouse model

*P. aeruginosa* strain PAO1 was grown to an optical density at 600 nm (OD_600_) of 1 in tryptic soy broth medium. Subsequently cells were washed twice with sterile PBS, and resuspended to a final concentration of 5 × 10^7^ CFU in 50 μL. To generate skin infection, female CD-1 mice (6 weeks old) were anesthetized with isoflurane and had their backs shaved. A superficial linear skin abrasion was made with a needle in order to damage the stratum corneum and upper layer of the epidermis. Five minutes after wounding, an aliquot of 50 μL containing 5 × 10^7^ CFU of bacteria in PBS was inoculated over each defined area containing the scratch with a pipette tip. One day after the infection, peptides were administered to the infected area. Animals were euthanized and the area of scarified skin was excised 2 and 4 days post-infection, homogenized using a bead beater for 20 min (25 Hz), and serially diluted for CFU quantification. Two independent experiments were performed with four mice per group in each case. Statistical significance was assessed using a two-way ANOVA. Animals were maintained in accordance with the *Guide for the Care and Use of Laboratory Animals* in an AAALAC-accredited facility. All procedures were approved by the MIT’s Institutional Animal Care and Use Committee (IACUC), protocol number 1016-064-19.

### CD spectroscopy

CD assays were carried out using JASCO J-815 spectropolarimeter equipped with a Peltier temperature controller (model PTC-423L/15). Measurements were recorded at 25 °C and performed in quartz cells of 1 mm path length between 195 and 260 nm at 0.2 nm intervals. Six repeat scans at a scan-rate of 50 nm min^−1^, 1 s response time and 1 nm bandwidth were averaged for each sample and for the baseline of the corresponding peptide-free sample. After subtracting the baseline from the sample spectra, CD data were processed with the Spectra Analysis software, which is part of Spectra Manager Platform.  Spectra were obtained in the following conditions, water (pH 7), sodium dodecyl sulfate (20 mM; pH 4, 7 or 10), dodecylphosphocholine (20 mM; pH 4) and 2,2,2-Trifluoroetanol 50% (v/v; pH 4). The peptide concentration was 33 and 38 μM for water and other systems, respectively. The relative helix content (*H*) according to the number of peptide bonds (*n*) was calculated from the ellipticity values at 222 nm^60^.

### NMR spectroscopy and structure calculations

The NMR sample was prepared by dissolving guavanin 2 in a micellar solution containing 100 mM of deuterated dodecylphosphocholine (DPC-d_38_) and 5% D_2_O at 1 mM concentration. The pH was adjusted to 4.0. All spectra were acquired at 25 °C on a Bruker Avance III 500 spectrometer equipped with a 5 mm triple resonance broadband inverse (TBI) probehead. Proton chemical shifts were referenced to sodium 2,2-dimethyl-2-silapentane-5-sulfonate (DSS) and water suppression was achieved using the pre-saturation technique. ^1^H-^1^H TOCSY experiment was recorded with 128 transients of 4096 data points, 256 t1 increments, and a spinlock mixing time of 80 ms. The ^1^H-^1^H NOESY was recorded with 64 transients of 4096 data points, 256 t1 increments, mixing time of 250 ms. Spectral width of 8012 Hz in both dimensions. ^1^H-^13^C HSQC experiment was acquired with F1 and F2 spectral widths of 8012 and 25152 Hz, respectively, were collected 256 t1 increments with 96 transients of 4096 points for each free induction decay. The experiment was acquired in an edited mode. All NMR data were processed using NMRPIPE^61^ and analyzed with NMR View^62^.

The structure calculations were performed with the XPLOR-NIH version 2.28 software by simulated annealing (SA) algorithm^63^. NOE intensities were converted into semi-quantitative distance restrains using the calibration by Hyberts et al.^64^. The angle restraints of phi and psi of the protein backbone dihedral angles were predicted based on analysis of ^1^H_α_ and ^13^C_α_ chemical shifts using the program TALOS+^65^. Several cycles of XPLOR were performed using standard protocols. After each cycle rejected restraints, side-chain assignments, NOEs, and dihedral violations were analyzed. Two hundred structures were calculated, and among them, the 20 lowest energy structures were submitted to XPLOR-NIH water refinement protocol^63^. The ensemble of the 10 lowest energy conformations was chosen to represent the solution structure ensemble of guavanin 2.

The restrictions used in structural calculations were analyzed by QUEEN program (Quantitative Evaluation of Experimental NMR Restraints). This program performs a quantitative assessment of the restrictions of the experimental NMR data. QUEEN checks and corrects possible assignments of errors by the analysis of the restrictions^66^. The stereochemical quality of the lowest energy structures was analyzed by PROCHECK^50^ and ProSA^49^. PROCHECK was used in order to check stereochemical quality of protein structure through the Ramachandran plot, where good quality models are expected to have more than 90% of amino acid residues in most favored and additional allowed regions. ProSA indicates the fold quality by means of the *Z*-score. The display, analysis, and manipulation of the three-dimensional structures were performed with the program MOLMOL^67^ and PyMOL (The PyMOL Molecular Graphics System, Version 1.8 Schrödinger, LLC).

### Solvation potential energy calculation

The solvation potential energy was measured for the ten lower energy NMR structures. Each structure was separated into a single pdb file. The conversion of pdb files into pqr files was perfomed by the utility PDB2PQR using the AMBER force field^68^. The grid dimensions for Adaptive Poisson-Boltzmann Solver (APBS) calculation were also determined by PDB2PQR. Solvation potential energy was calculated by APBS^69^. Surface visualization was performed using the APBS plugin for PyMOL.

### Data availability

All data are available from the authors upon reasonable request. The protein structure is deposited in the Protein Data Bank with ID 5V1E and BMRB 20163.

## Electronic supplementary material

Supplementary Information
