# Supplementary material for: In silico optimization of a guava antimicrobial peptide enables combinatorial exploration for peptide design
Source: Nat Commun. 2018 Apr 16;9:1490. doi: 10.1038/s41467-018-03746-3 (PMC5902452; doi:10.1038/s41467-018-03746-3)
Supplement: Supplementary file 1 — Supplementary Information [file 41467_2018_3746_MOESM1_ESM.pdf]

Supplementary Information

In silico optimization of a guava antimicrobial peptide enables combinatorial exploration for peptide design

William F. Porto[a,b,c], Luz Irazazabal[a,d], Eliane S. F. Alves[d], Suzana M. Ribeiro[b], Carolina O. Matos[e], Állan S. Pires[a], Isabel C. M. Fensterseifer[a,d], Vivian J. Miranda[a], Evan F. Haney[f], Vincent Humblot[g], Marcelo D. T. Torres[h,i,j], Robert E. W. Hancock[f], Luciano M. Liao[e], Ali Ladram[k], Timothy K. Lu[h,i], Cesar de la Fuente-Nunez[h,i]* and Octavio L. Franco[a,b,d]*

[a] Centro de Análises Proteômicas e Bioquímicas, Pós-Graduação em Ciências Genômicas e Biotecnologia Universidade Católica de Brasília, Brasília-DF, Brazil.

[b] S-Inova Biotech, Pós-graduação em Biotecnologia, Universidade Católica Dom Bosco, Campo Grande, MS, Brazil.

[c] Porto Reports, Brasília-DF, Brazil

[d] Molecular Pathology Post-graduate Program, University of Brasília, Brasília, Distrito Federal, Brazil.

[e] Laboratório de RMN, Instituto de Química, Universidade Federal de Goiás, Goiânia, GO, Brazil.

[f] Centre for Microbial Diseases and Immunity Research, University of British Columbia, 2259 Lower Mall Research Station, Vancouver, British Columbia, Canada V6T 1Z4.

[g] Sorbonne Universités, UPMC Univ Paris 06, UMR 7197, Laboratoire de Réactivité de Surface (LRS), F-75005, Paris, France.

[h] Synthetic Biology Group, MIT Synthetic Biology Center; The Center for Microbiome Informatics and Therapeutics; Research Laboratory of Electronics, Department of Biological Engineering, and Department of Electrical Engineering and Computer Science, Massachusetts Institute of Technology, Cambridge, MA, United States of America.

[i] Broad Institute of MIT and Harvard, Cambridge, Massachusetts, United States of America.

[j] Centro de Ciências Naturais e Humanas, Universidade Federal do ABC, Santo André, São Paulo, Brazil.

[k] Sorbonne Universités, UPMC Univ Paris 06, CNRS, Institut de Biologie Paris-Seine (IBPS), Biogenèse des Signaux Peptidiques (BIOSIPE), F-75005 Paris, France.

*Corresponding authors:

Cesar de la Fuente-Nunez - cfuente@mit.edu, Phone number: +1 617 324 4227

Octavio Luiz Franco - ocfranco@gmail.com, Phone number: +55 61 34487167/ +55 61 34487220
Fax number: + 55 61 33474797, website: http://www.capb.com.br

Supplementary Figures

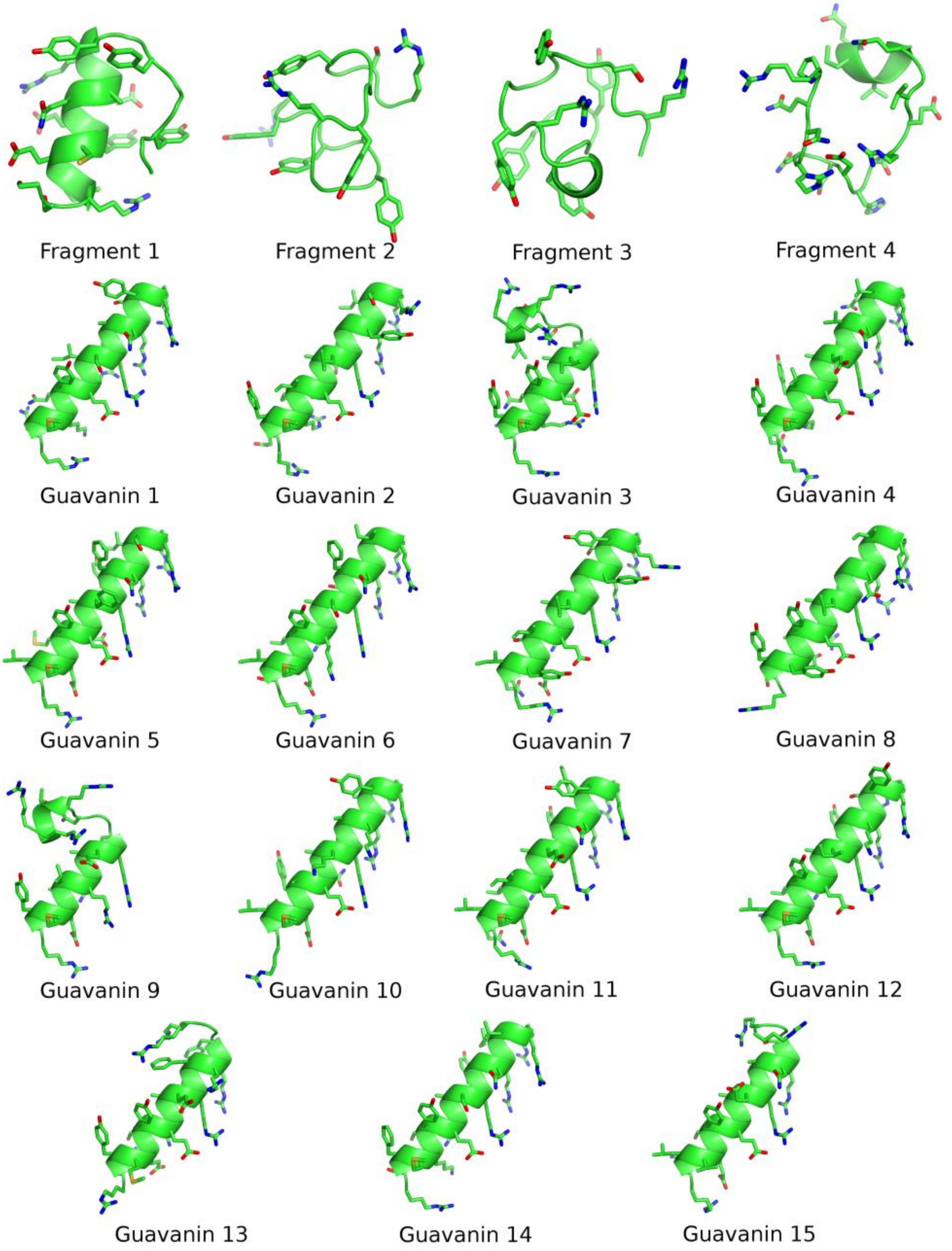

Supplementary Figure 1. *Ab initio* models of the 4 fragments of Pg-AMP1 (Fragments 1-4) and the 15 best fitness designed guavanins. Fragments 1 to 4 represents the best α-helical propensity, higher net charge, hydrophobicity and hydrophobic moment, respectively. Their physicochemical properties are detailed on Supplementary Table 3. The four fragments present unusual predicted structures (Overall G-factors < -0.5). From guavanins, 13 out of 15 were predicted to be in 100% of α-helical structure. Guavanins 3 and 9 were predicted to have a loop in the C-terminal region, which is also considered unusual (Overall G-factors < -0.5). The model assessments are summarized in Supplementary Table 2.

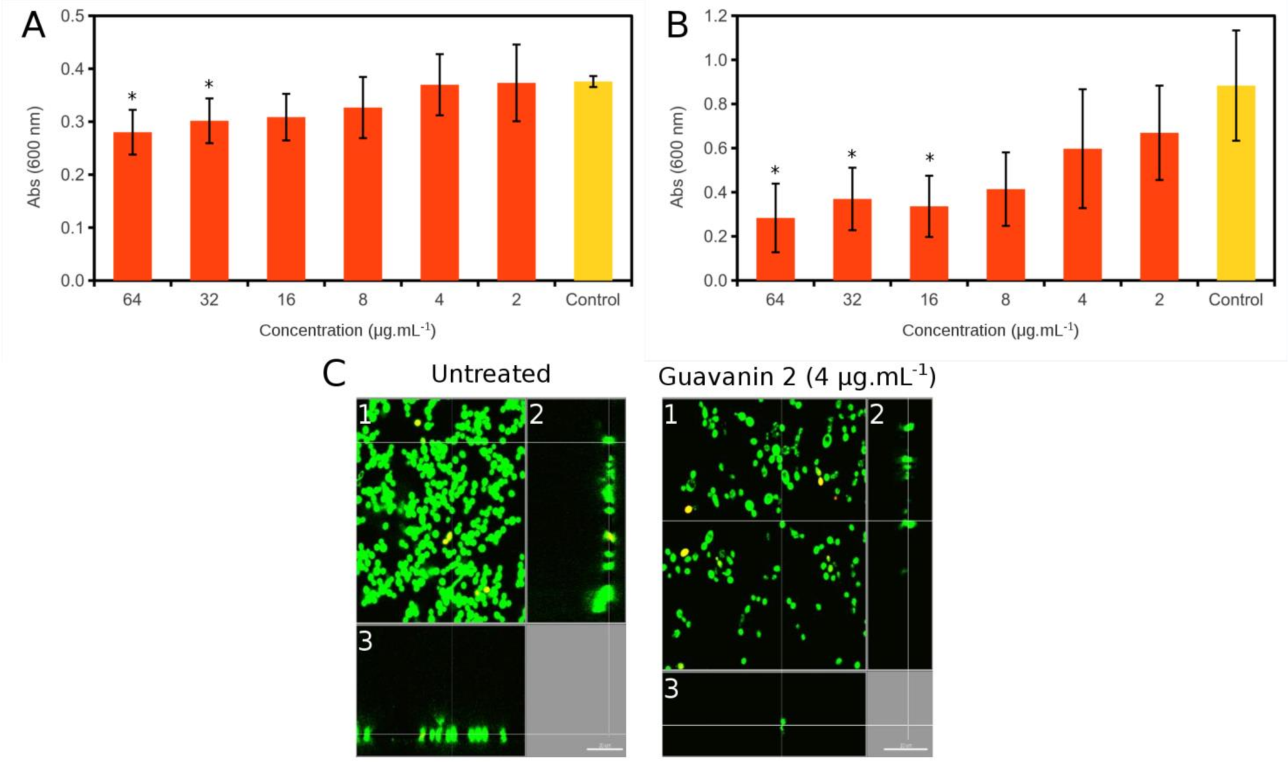

Supplementary Figure 2. Antibiofilm activity of guavanin 2 against *C. albicans*. (A) The peptide was not able to eliminate the planktonic cells, however, starting at 16 µg.mL^{-1}, it shows some effect with statistical significance ($p<0.05$, Student's t-test). (B) Guavanin 2 was able to inhibit the formation of biofilm starting at 32 µg.mL^{-1} ($p<0.05$, Student's t-test). The error bars indicates the standard deviation of three experimental replicates. (C) Preformed biofilm disruption by guavanin 2 (4µg.mL^{-1}). The biofilms were grown in a flow cell system during 48h with BM2 media. After that, the peptide was applied during 24 h. Candida biofilms were stained green with Syto-9 which stains all cells and red with propidium iodide which stains dead cells red (merge shows as yellow to red). Reconstructions from the xy, yz and xz dimensions are represented in panels 1, 2 and 3, respectively. Scale bar = 40 and 30 µm for untreated and treated samples, respectively.

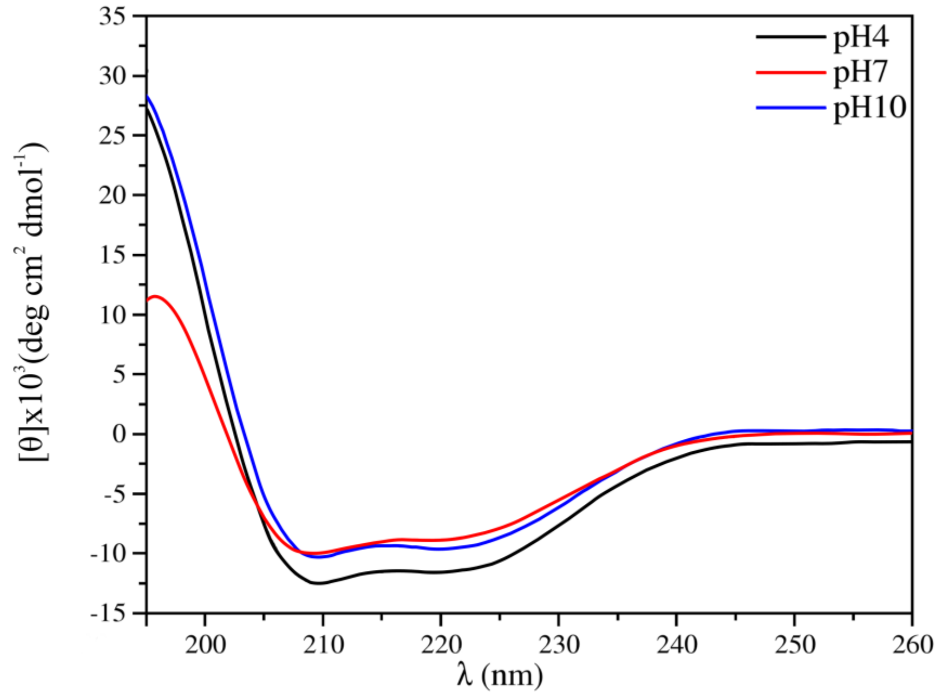

Supplementary Figure 3. CD spectra of guavanin 2 at 25 °C in SDS (20mM) and pH 4, pH 7 and pH 10. The α-helical structure of guavanin 2 is more stable in acidic pH than neutral or alkaline one. It may occur due to the neutralization of arginine residues, avoiding the repulsion among them and stabilizing the structure.

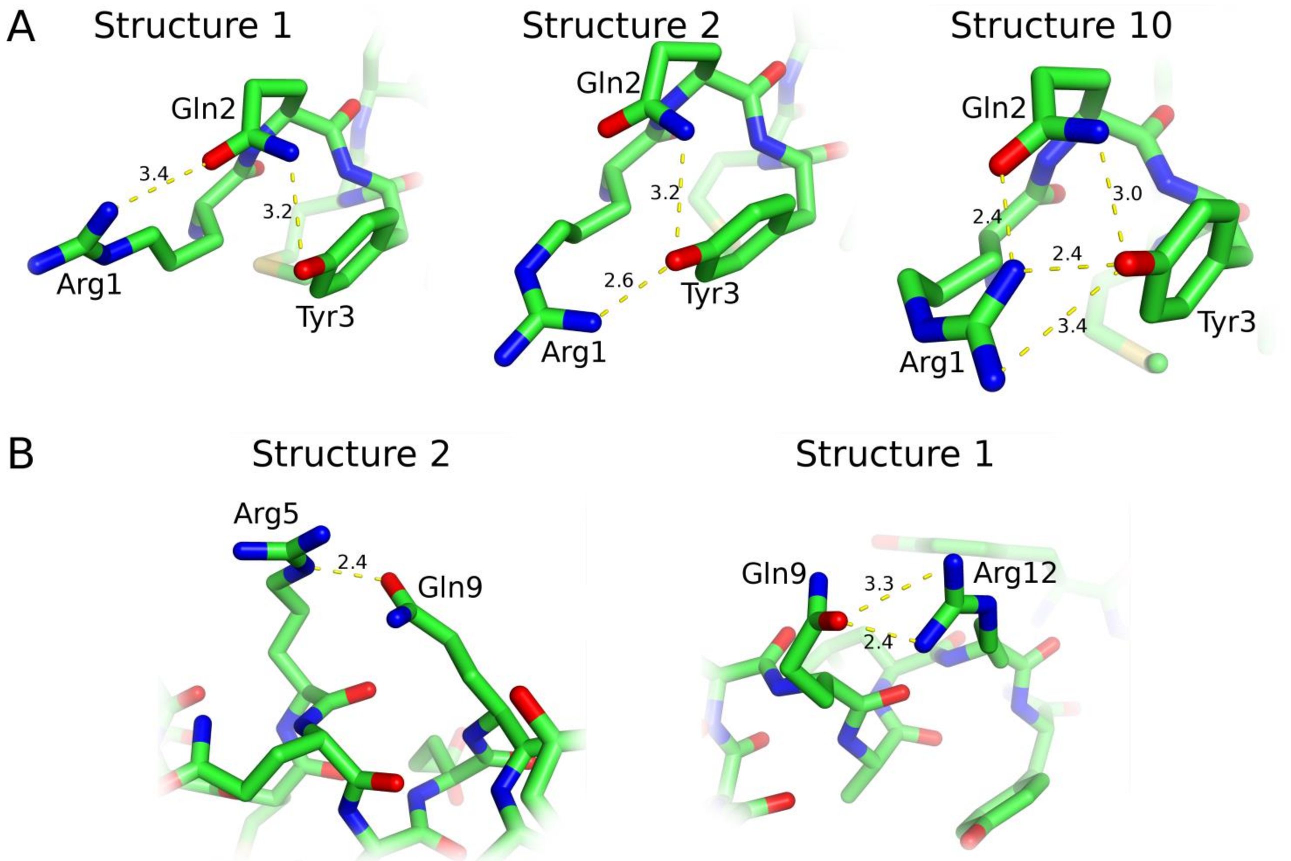

Supplementary Figure 4 – Hydrogen bonding network involving side chains of guavanin 2. (A) The N-Terminal region is stabilized by the residues Arg^1, Gln^2 and Tyr^3, which interact with each other and whose positions vary depending on the structure evaluated from the NMR ensemble; the three possibilities observed are represented by structures 1, 2 and 10. (B) The Gln^9 side chain interacts with surrounding Arg residues (Arg^5 and Arg^{12}), the two possibilities observed are represented by structures 1 and 2.

Supplementary Tables

Supplementary Table 1. The best sequences of each parallel run of our genetic algorithm.

Guavanin	Sequence	Fitness	Guavanin	Sequence	Fitness
1	RRGMKQYERISRDANRSYRR	0.393	51	RAYMECLEQAERYGNRAYRR	0.324
2	RQYMRQIEQALRYGYRISRR	0.390	52	RQVMETYEQLERYGNRSARR	0.323
3	RKYMRQYEEAIRDGNRSIRR	0.390	53	RQIRECYEQASRYGNRSYRR	0.323
4	RQYMRYLEQAERYVNRNLRR	0.389	54	RQYMEVYEQAERAGNRVYRR	0.322
5	RKLMEMYEEAFRYFNRISRR	0.386	55	RSYMEQYEQAFRRGNRSYRR	0.322
6	RSIMELYKQASRSFNRGIRR	0.379	56	RHFMECYEQASRDGNRSLRR	0.321
7	RQIYESIEQALRRGYRSYRR	0.378	57	RKAMEQYEEAERDGARSYRR	0.321
8	RSYYEAYERALRKGQRGIRR	0.371	58	RQYMKGYEQAERHAYRSYRR	0.320
9	RAYMEALRQAERLGNRTARR	0.370	59	RQYMEQAEQAERDGNRSVRR	0.319
10	RYLMEYAEQAKRDAKRAYRR	0.370	60	RSIMEYYEQIERDGNRSYRR	0.318
11	RQLMELIEQAERYGNRFYRR	0.368	61	RYLKECYEQASRIGYRGLRR	0.318
12	RKLMELYEQAIRYGKRSYRR	0.364	62	RQGMEAYEQAERLGNRGIRR	0.318
13	RRYMECYEQAERYFRRFGRR	0.362	63	RQYMECYKQIYRYGNRSYRR	0.318
14	RSFMKCYEQASRYGNRILRR	0.362	64	RSYREYAEQALRYGNRGYRR	0.317
15	RKLVECYERAERDANRSGRR	0.361	65	RSGMEYYKQAFRAGYRVTRR	0.316
16	RQLMECYEQAARRGARSYRR	0.359	66	RSAMECYEKAERYWYRGSRR	0.316
17	RYMMKIYEQAERYFNRVGRR	0.359	67	RSYMECYEQASRKGNRSIRR	0.316
18	RRYYEQLEQASRKGNRGFRR	0.356	68	RQYMELYEQAMRYGNRGYRR	0.315
19	RSVMEQYEQAARDAYRSARR	0.355	69	RQYIECYEQAARYGKRGYRR	0.315
20	RQYMECIEKALRDGYRSYRR	0.352	70	RQWAEYYEQLERYGNRSYRR	0.315
21	RYYMKCYKQAARYIYRGYRR	0.351	71	RSYMEAYEQASRDGYRLYRR	0.314
22	RSAYEYYRRAYRDGNRGYRR	0.351	72	RQYMEQYEQFERAGNRVYRR	0.314
23	RYGMRQFEQASRDGNRSFRR	0.349	73	RYYMEYYEKASRYGNRGIRR	0.313
24	RKGYRGYEQALRYGKRYGRR	0.347	74	RYYMEYYEQLERYGNRLYRR	0.312
25	RYGMRCLEEALRYGNRGYRR	0.347	75	RQYMECYEQAARYGNRSYRR	0.309
26	RQYREIIEQARRVGNRGARR	0.347	76	RQYMEIYEQASRYGNRSYRR	0.307
27	RQGMEVYERASRQGNRSLRR	0.346	77	RQYMEQYEQAMRDGNRGYRR	0.306
28	RRIMEQYEEAERDGNRVYRR	0.346	78	RQYMEYYEQFSRLGNRSYRR	0.305
29	RQVMEAYEQFYRDGNRAYRR	0.343	79	RSGMKVYEQAERYGNRSYRR	0.304
30	RQLMEQYEQAYRYAARGYRR	0.343	80	RSAMECYEKASRDGNRGSRR	0.304
31	RYIMEIYEQAIRKGNRSYRR	0.341	81	RYYKEYYEKAERIGNRGYRR	0.304
32	RKYMELYEKASRRGYRGYRR	0.338	82	RSYMECYEQAFRYGKRSSRR	0.303
33	RQYLEQYENAERYIYRAYRR	0.333	83	RQYMECYKQAERYGNRGYRR	0.302
34	RQYMKCYEQAYRYGRRGYRR	0.332	84	RSVMEYYEQAYRYGNRGSRR	0.301
35	RQYAEQYEEAIRDGNRSVRR	0.331	85	RQGMEAYEQAERYGNRSYRR	0.298
36	RSYMEMLEQIERYGNRVGRR	0.330	86	RAYQEAYEQAYRDGNRSYRR	0.298
37	RQYMEFVEQAERYGRRGSRR	0.330	87	RSYMEQYEQASRKGYRSYRR	0.298
38	RSYMEQYEEAIRRGYRSYRR	0.329	88	RSYAECYEQISRYGNRGYRR	0.298
39	RQYMKYYEEAERYGNRAYRR	0.328	89	RSYMEAYEQAERYGNRGYRR	0.296
40	RAYMEYYEQFYRMGKRASRR	0.328	90	SQRVEQYVRRLYDDYRNYMR	0.295
41	RQYMEQVEQALRDGYRSGRR	0.327	91	RSYIEQYEQLERDGARSYRR	0.294
42	RSYMESIEQALRIGNRSYRR	0.327	92	SQRLERYVERSFDDYRKSGR	0.292
43	RSYMEIYEQASRAGNRAYRR	0.327	93	RSYMEYYEQASRDGARGYRR	0.290
44	RQYMEYYEQVFRAGYRSARR	0.327	94	SKRVGQGVERSYKKYRNYIR	0.272
45	RYYMECYEQAVRYGRRWYRR	0.325	95	GQRVEQLVERYGDDLRNSVR	0.267
46	RQGMECYEQALRYGQRGIRR	0.325	96	YQRVEQYVQRSYDAYRNYAR	0.259
47	RSFMEQGEQAFRDGYRMYRR	0.325	97	SQRVEQYVERYADGYRNYLR	0.258
48	RKYMEIYEKASRYGNRSYRR	0.325	98	YQRVEQYVQRYHDDLRNYSR	0.256
49	RQYKEAYEEIYRYGNRMGRR	0.325	99	YQRVEQYVQRSYDDYRNVGR	0.245
50	RRYMECYEQAERDGNRMYRR	0.324	100	TQRVEQYVERSSDKYRNLGR	0.245

Supplementary Table 2. Structural assessments of ab initio models of the 4 Pg-AMP1 fragments and 15 best fitness guavanins

Peptide	DOPE	ProSA (Z-Score)	Ramachandran Plot (%)		G-Factor
			Favored Regions	Allowed Regions	
Fragment 1[a]	-1228.738	-1.22	100	0	-0.99
Fragment 2 [a,b]	-337.424	-1.96	28.6	57.1	-2.57
Fragment 3 [a,b]	-489.954	-1.27	71.4	14.3	-2.26
Fragment 4 [a,b]	-703.175	-1.18	78.6	14.3	-1.91
Guavanin 1	-1644.390	-1.12	100	0	-0.09
Guavanin 2	-1891.091	-0.73	100	0	-0.13
Guavanin 3 [a]	-1519.247	-0.99	94.1	5.9	-0.80
Guavanin 4	-1950.491	-1.25	100	0	0.10
Guavanin 5	-1902.878	-1.00	100	0	0.01
Guavanin 6	-1633.499	-0.48	100	0	-0.26
Guavanin 7	-1779.689	-1.08	100	0	-0.17
Guavanin 8	-1563.839	-1.14	100	0	-0.28
Guavanin 9[a]	-1595.297	-1.6	94.1	5.9	-0.76
Guavanin 10	-1825.547	-1.3	100	0	0.18
Guavanin 11	-1881.204	-1.08	100	0	0.03
Guavanin 12	-1851.237	-1.23	100	0	-0.04
Guavanin 13	-1661.289	-1.61	100	0	-0.26
Guavanin 14	-1741.938	-0.79	100	0	-0.04
Guavanin 15	-1633.659	-1.59	100	0	-0.26

[a] unusual structure according to G-Factor

[b] Structures with at least five gly or pro residues, which are not taken into account for Ramachandran Plot analysis.

Supplementary Table 3 – Physicochemical properties and biological activity assessments of Pg-AMP1 fragments, guavanins 1-15 and magainin 2 (positive peptide control).

Peptide	Sequence*	F	M	H	A	Q	MIC(μg.mL^{-1})**	Hemolysis (μg.mL^{-1})***
Fragment 1 (α-helix)	SSRMECYEQAERYGYGGYGG	n/a	0.089	-0.262	**0.553**	0	>200	>200
Fragment 2 (net charge)	RYGYGGYGGGRYGGGYGSGR	n/a	0.100	-0.190	0.739	**+4**	200	100
Fragment 3 (hydrophobicity)	YGYGGYGGGRYGGGYGSGRG	n/a	0.027	**-0.092**	0.779	+3	>200	>200
Fragment 4 (hydrophobic moment)	GQPVGQGVERSHDDNRNQPR	**n/a**	**0.300**	-0.503	0.829	+2	>200	>200
Guavanin 1	RRGMKQYERISRDANRSYRR	0.393	0.589	-0.773	0.379	+7	200	>200
Guavanin 2	RQYMRQIEQALRYGYRISRR	0.390	0.572	-0.552	0.360	+6	6.25	>200
Guavanin 3	RKYMRQYEEAIRDGNRSIRR	0.390	0.587	-0.664	0.384	+5	>200	>200
Guavanin 4	RQYMRYLEQAERYVNRNLRR	0.389	0.560	-0.627	0.350	+5	100	>200
Guavanin 5	RKLMEMYEEAFRYFNRISRR	0.386	0.552	-0.479	0.345	+4	100	>200
Guavanin 6	RSIMELYKQASRSFNRGIRR	0.379	0.568	-0.477	0.380	+6	100	>200
Guavanin 7	RQIYESIEQALRRGYRSYRR	0.378	0.562	-0.574	0.373	+5	200	>200
Guavanin 8	RSYYEAYERALRKGQRGIRR	0.371	0.558	-0.598	0.371	+6	100	>200
Guavanin 9	RAYMEALRQAERLGNRTARR	0.370	0.516	-0.553	0.298	+5	>200	>200
Guavanin 10	RYLMEYAEQAKRDAKRAYRR	0.370	0.496	-0.600	0.275	+5	200	>200
Guavanin 11	RQLMELIEQAERYGNRFYRR	0.368	0.544	-0.489	0.368	+3	>200	>200
Guavanin 12	RKLMELYEQAIRYGKRSYRR	0.364	0.526	-0.544	0.346	+6	25	>200
Guavanin 13	RRYMECYEQAERYFRRFGRR	0.362	0.545	-0.658	0.383	+5	25	>200
Guavanin 14	RSFMKCYEQASRYGNRILRR	0.362	0.551	-0.498	0.395	+6	12.5	>200
Guavanin 15	RKLVECYERAERDANRSGRR	0.361	0.546	-0.680	0.380	+4	200	>200
Magainin 2	GIGKFLHSAKKFGKAFVGEIMNS	0.168	0.286	-0.036	0.489	+5	100	>200

*All peptides are amidated.
** MICs evaluated on SPOT-synthesized peptide samples of unpurified crude synthetic peptide (~70% purity) against the bioluminescent engineered strain of the Gram-Negative bacteria *P. aeruginosa*.
***100% of hemolysis was not observed.
F, fitness; μ, hydrophobic moment; H, hydrophobicity; α, α-helix propensity; Q, net charge.

Supplementary Table 4. Amino acid probability distributions. This distribution was based on the frequency of occurrence of each amino acid according to the Antimicrobial Peptides Database (APD – Accessed on April, 2013) (36). Cysteine, aspartic acid, glutamic acid, glycine and proline residues were removed from the set and the probability distribution was adjusted for remaining residues.

**Residue**	**Distribution (%)**	**Residue**	**Distribution (%)**	**Residue**	**Distribution (%)**
A	11.092	L	11.869	S	8.281
F	5.624	M	1.597	T	6.132
H	2.925	N	5.341	V	8.111
I	8.563	Q	3.207	W	2.247
K	13.494	R	7.984	Y	3.533
